# A link between pH homeostasis and colistin resistance in bacteria

**DOI:** 10.1038/s41598-021-92718-7

**Published:** 2021-06-24

**Authors:** Pradip R. Panta, William T. Doerrler

**Affiliations:** Department of Biological Sciences, Louisiana State University, Baton Rouge, LA USA

**Keywords:** Chemical biology, Microbiology

## Abstract

Colistin resistance is complex and multifactorial. DbcA is an inner membrane protein belonging to the DedA superfamily required for maintaining extreme colistin resistance of *Burkholderia thailandensis*. The molecular mechanisms behind this remain unclear. Here, we report that ∆*dbcA* displays alkaline pH/bicarbonate sensitivity and propose a role of DbcA in extreme colistin resistance of *B. thailandensis* by maintaining cytoplasmic pH homeostasis. We found that alkaline pH or presence of sodium bicarbonate displays a synergistic effect with colistin against not only extremely colistin resistant species like *B. thailandensis* and *Serratia marcescens*, but also a majority of Gram-negative and Gram-positive bacteria tested, suggesting a link between cytoplasmic pH homeostasis and colistin resistance across species. We found that lowering the level of oxygen in the growth media or supplementation of fermentable sugars such as glucose not only alleviated alkaline pH stress, but also increased colistin resistance in most bacteria tested, likely by avoiding cytoplasmic alkalinization. Our observations suggest a previously unreported link between pH, oxygen, and colistin resistance. We propose that maintaining optimal cytoplasmic pH is required for colistin resistance in a majority of bacterial species, consistent with the emerging link between cytoplasmic pH homeostasis and antibiotic resistance.

## Introduction

Colistin is a cationic antimicrobial peptide (CAMP) belonging to the polymyxin antibiotic family, which was first isolated from a Gram-positive soil bacteria *Bacillus polymyxa*^1^. A rise in multidrug resistance among bacterial pathogens and a lack of new antimicrobial drugs have led to renewed interest in reviving older antimicrobial agents such as polymyxin^2^. Despite its nephrotoxicity and neurotoxicity, polymyxin still remains one of the most effective CAMPs and is presently classified as “Reserve Group” of antibiotics, that can be used as a last resort antibiotic against pathogens resistant to most commonly used antibiotics^3–5^. However, the escalating use of colistin along with the rise in colistin resistance among several bacterial pathogens and the global spread of *mobilized colistin resistance* (*mcr*) genes are greatly worrisome^6, 7^.

Unlike most Gram negative bacteria, *Burkholderia* spp. display extremely high intrinsic polymyxin resistance^8^ and remain as important model system for understanding the molecular mechanism behind such a high level resistance. Several studies in *Burkholderia* have revealed that the extreme polymyxin resistance is likely multifactorial consisting of major and minor determinants of resistance^9–14^. One such unique genetic determinant of high level colistin resistance of *Burkholderia thailandensis* is DbcA (BTH_I1321), a highly conserved inner membrane protein belonging to DedA superfamily^15^. *B. thailandensis* DbcA is required for efficient lipid A modification with Ara4N and high level colistin resistance^15, 16^. DedA proteins have also been reported to be required for polymyxin and/or CAMP resistance of *Salmonella enterica*^17^, *Neisseria meningitidis*^18^, *Klebsiella pneumoniae*^19^, and *Enterobacter cloacae*^20^. The DedA family of membrane proteins is widely distributed in nature, found in all domains of life, whose physiological function remains unknown. Studies in *Escherichia coli* have shown that redundant DedA family proteins YqjA and YghB are required for a number of proton motive force dependent processes including twin arginine transport (TAT) dependent protein export and cell division^21^, antibiotic resistance dependent on efflux pump activity^22^, and survival at alkaline pH^23^.

While the exact mechanism of colistin killing is still unclear, there are many models proposing its mode of action. Colistin is polycationic and amphipathic in nature. These properties allow colistin to be electrostatically attracted to negatively charged phosphates of lipid A, thereby displacing divalent cations such as calcium and magnesium ions that normally bridge adjacent lipid A molecules and stabilize the outer membrane^24^. Disruption of outer membrane permeability promotes the uptake of the colistin itself, hence the term “self-promoted uptake”^24^. What happens next is not completely understood. It is believed that polymyxin can further disrupt the inner membrane phospholipid bilayer and induce the formation of pores, leading to leakage of cytoplasmic contents, and ultimately cell death by lysis^25^. A recent finding that polymyxin can target LPS in the inner membrane of polymyxin sensitive *E. coli* supports the notion that the bactericidal activity of polymyxin requires the disruption of inner membrane and cytoplasmic entry^26^. Polymyxin can increase cytoplasmic membrane permeability, as assessed by membrane depolarization in *E. coli* and *Pseudomonas aeruginosa*; however, bactericidal activity of polymyxin does not require membrane depolarization^27, 28^. While membrane depolarization induced by a protonophore CCCP has been shown to sensitize many Gram-negative species to polymyxin^15, 29–32^, membrane hyperpolarization has also been proposed to be responsible for increased polymyxin sensitivity in *E. coli* ∆*phoP*^33^, and *Staphylococcus aureus* ∆*atpA*^34^. Therefore, the role of membrane potential in polymyxin resistance is not clearly understood. PhoP is a broadly conserved response regulator of a two component system PhoPQ involved in many important processes such as proper maintenance of periplasmic redox state^35–37^, activation of type VI secretion system in intra and inter species bacterial competition through ROS production^38^ and polymyxin resistance through induction of genes involved in LPS modifications and maintenance of reversed membrane potential (more positive inside)^33^. *AtpA* encodes the alpha subunit of ATP synthase^34^. Our earlier report shows that maintaining membrane potential and extreme colistin resistance by *B. thailandensis* E264 and *∆dbcA* is dependent upon the pH of the media^16^. Therefore, we measured colistin MIC of different bacteria grown at different pH to determine the effect of external pH in colistin toxicity. Sodium bicarbonate has been reported to sensitize *S. aureus* to polymyxin by alkalinizing the cytoplasm and altering the PMF^39^. Here, we explored the effect of sodium bicarbonate on colistin susceptibility of different bacterial species.

Another hypothesis of bactericidal activity of polymyxin is through oxidative stress. There are many reports suggesting that polymyxin can induce oxidative stress and cause cell death in many Gram-negative bacteria^40–44^. However, a few reports do not support this mechanism of killing by polymyxin, assessed by the equal killing by polymyxin at both aerobic and anaerobic conditions^45^ and the ability of colistin to effectively kill metabolically inactive and hypoxic subpopulation within the internal regions of biofilm in *P*. *aeruginosa*^46^. Whether bactericidal activity of colistin is dependent on oxygen is still unclear. We explored colistin resistance of different bacteria under normal, hypoxic, and anoxic conditions, and propose a link between pH and oxygen. We also show the dependence of colistin resistance on fermentable carbon source such as glucose and connect the effect of glucose back to pH homeostasis. Our study in *Burkholderia ∆dbcA* reveals a link between pH and colistin resistance not only in *B. thailandensis*, but also in other bacterial species. This study reveals an unexpected complexity of colistin resistance in both Gram-negative and Gram-positive bacteria.

## Results

### *B. thailandensis *∆*dbcA* is sensitive to alkaline pH

**Figure 1:**
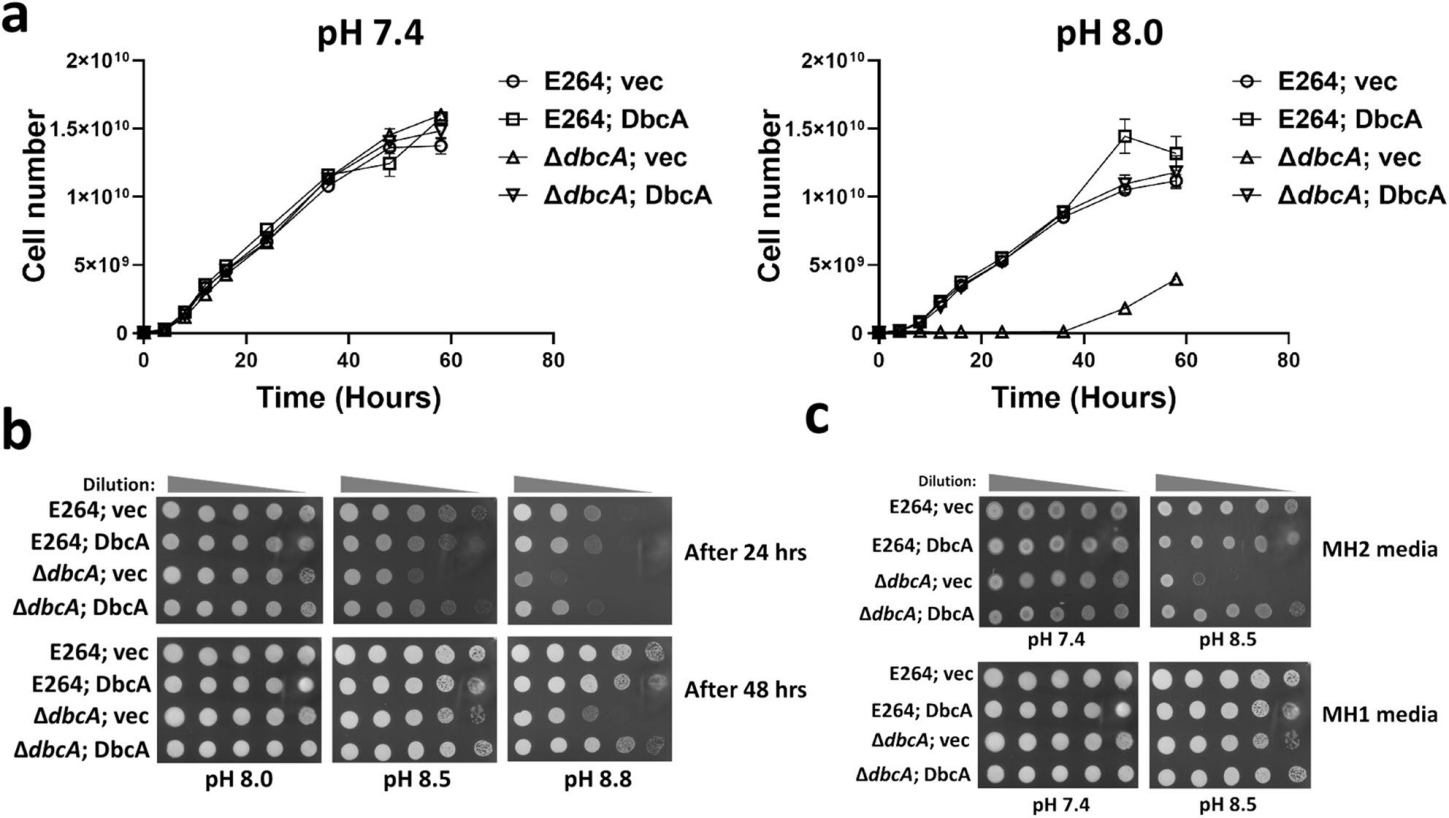
Alkaline pH sensitivity of *B. thailandensis* ∆*dbcA.* (**a**) Growth rate of *B. thailandensis* strains (E264; vec, E264; DbcA, ∆*dbcA*; vec, and ∆*dbcA*; DbcA) at different times in Mueller Hinton broth (MH1) with 100 µg/ml Tmp. The error bars indicate standard deviations of three biological replicates. (**b**) Spot assay of E264; vec, E264; DbcA, ∆*dbcA*; vec, and ∆*dbcA*; DbcA on MH1 agar media. 1:10 dilutions of indicated strains were spotted on NaOH adjusted MH1 media with 100 µg/ml Tmp and 0.001% rhamnose. Plates were analyzed after 24 or 48 h at 37 °C. (**c**) Comparison of alkaline pH sensitivity of *B. thailandensis* ∆*dbcA* in MH2 with MH1 agar media. MH2 contains ~ 20 to 25 mg/l of calcium and ~ 10 to 12.5 mg/l of magnesium, whereas MH1 contains no added calcium and magnesium salts. The pH was adjusted by either HCl or NaOH. Plates were analyzed after 48 h of growth at 37 °C.

In light of the sensitivity of *E. coli* ∆*yqjA* to alkaline pH^23^, we tested whether *B. thailandensis* **∆***dbcA* is also alkaline pH sensitive. In our previous report, we found that **∆***dbcA* is more sensitive to divalent cations such as calcium and magnesium, and **∆***dbcA* also showed greater sensitivity to colistin in MH2 media compared to MH1 media^16^. Therefore, we looked at the alkaline pH sensitivity of **∆***dbcA* in MH1 broth to exclude the effects of divalent cations. Figure 1a shows that **∆***dbcA* has a growth defect at pH 8.0 compared to pH 7.4. However, on MH1 agar plates, the alkaline pH sensitivity of **∆***dbcA* requires pH 8.5 and above (Fig. 1b). **∆***dbcA* is also more sensitive to alkaline pH in presence of divalent cations as shown in the Fig. 1c.

### External Alkaline pH can sensitize majority of bacteria tested to colistin

**Figure 2:**
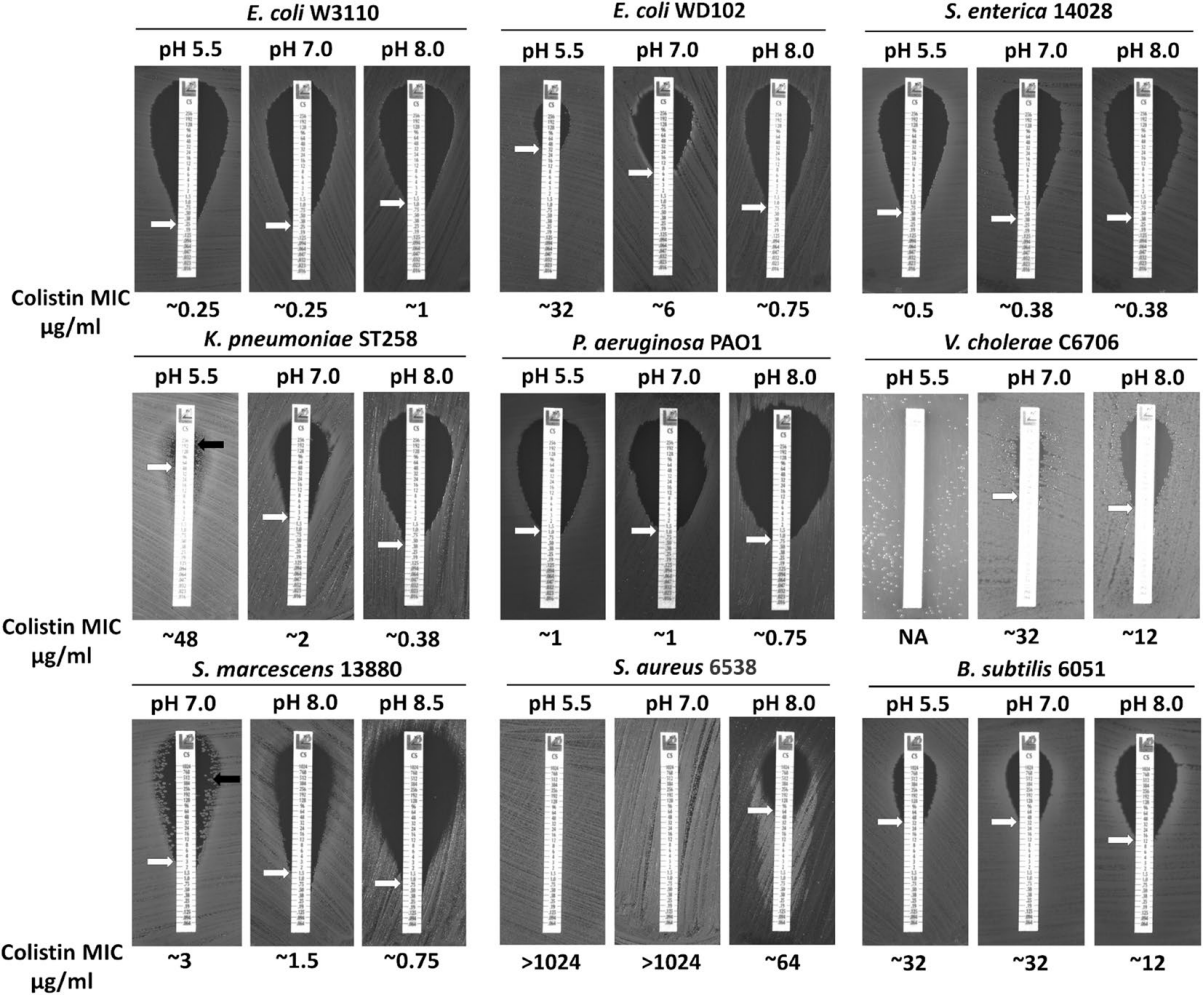
pH dependent colistin resistance of different bacterial species. Minimal inhibitory concentration (MIC) was determined for indicated strains on LB agar plates. The pH was adjusted with 100 mM MES for pH 5.5 and 100 mM Tris for pH 7.0, 8.0, and 8.5. Plates were analyzed after 24 h of growth at 37 °C. Approximate MICs are denoted by white arrows. Black arrows represent heteroresistant colonies of *S. marcescens or K*. *pneumoniae*.

We previously showed that alkaline pH of the media can sensitize extremely colistin resistant bacteria like *B. thailandensis* to colistin^16^. We hypothesized that alkaline pH could also sensitize other bacterial species to colistin. We used different bacteria with different colistin resistance profiles and measured the colistin MIC at different pH. In a majority of bacteria including Gram-positive species such as *S. aureus* and *B. bacillus*, the colistin MIC decreased as the pH of the media increased (Fig. 2). Surprisingly, in colistin sensitive *E. coli* W3110 the colistin MIC slightly increased in alkaline pH media compared to the acidic media (Fig. 2). The colistin MIC of *Serratia marcescens*, on plate was ~ 3 µg/ml at pH 7.0 agar media. However, the MIC of *S. marcescens* in liquid media is more than 500 µg/ml (see Supplementary Fig. S1 online). The appearance of other colonies represented by a single black arrow (Fig. 2) are colistin heteroresistant colonies of *S. marcescens*^32^. We propose that these heteroresistant colonies of *S. marcescens* are responsible for the extreme colistin resistance of *S. marcescens* observed in liquid media. We observed that these heteroresistant colonies of *S. marcescens* do not appear as the pH of the media increased (Fig. 2). Moreover, alkaline pH appears to increase colistin sensitivity of isolated heteroresistant *S. marcescens* clones (see Supplementary Fig. S1 online). We also observed the appearance of heteroresistant colonies of *K. pneumoniae*^47^ at pH 5.5 agar plates that disappear at pH 7.0 and pH 8.0 (Fig. 2). These findings suggest that the heteroresistance to colistin is also dependent on the pH of the media.

### Sodium bicarbonate can sensitize most bacteria to colistin

**Figure 3:**
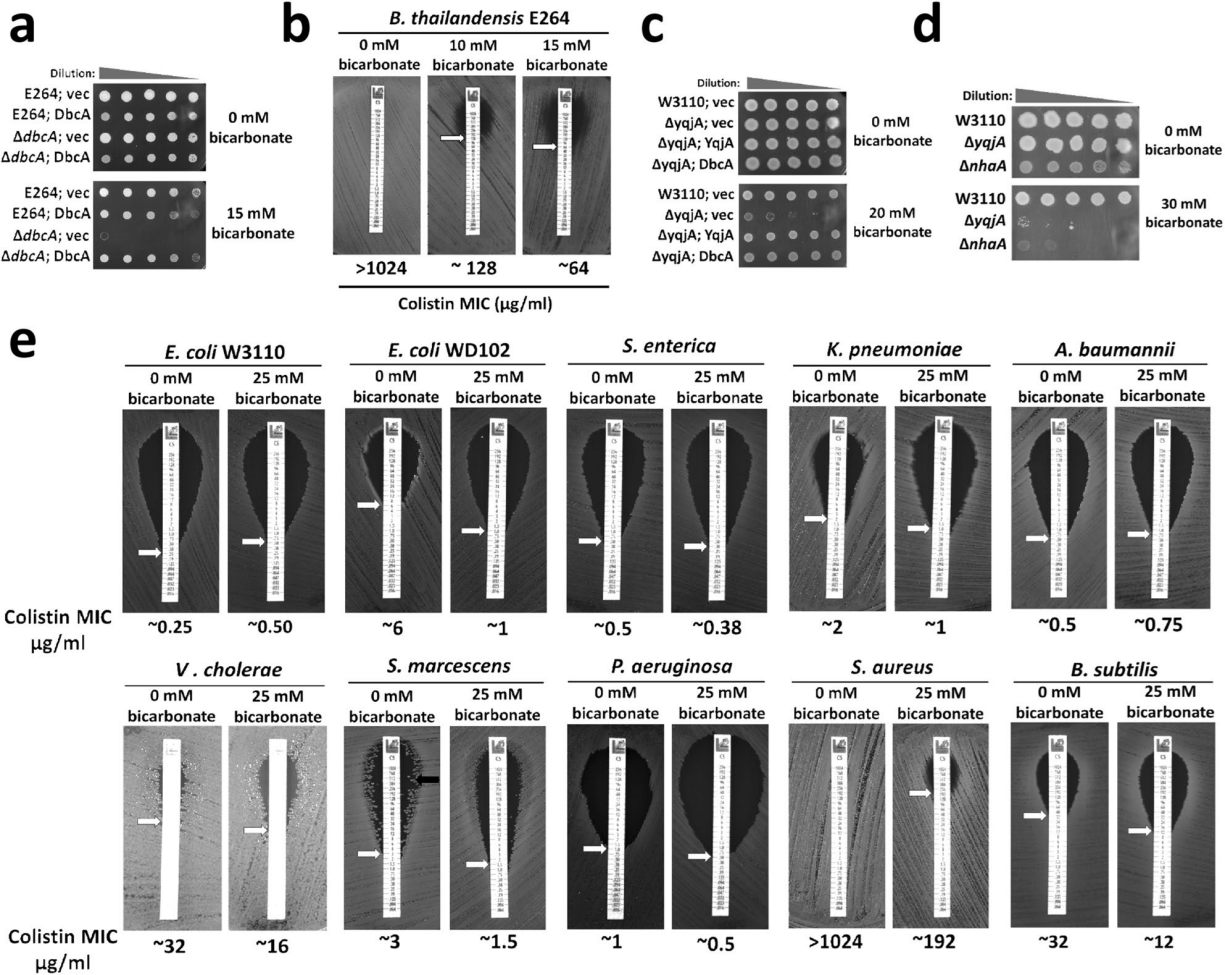
Effect of bicarbonate on colistin resistance. (**a**) Bicarbonate sensitivity of *B. thailandensis* ∆*dbcA*. Spot assay of *B. thailandensis* strains (E264; vec, E264; DbcA, ∆*dbcA*; vec, and ∆*dbcA*; DbcA) on MH2 agar media with 100 µg/ml Tmp, 0.002% rhamnose, and different concentrations of sodium bicarbonate as indicated. (**b**) Reduction in colistin MIC of *B. thailandensis* E264 by bicarbonate. The MIC was determined on MH2 agar plates with different concentrations of sodium bicarbonate as indicated. Approximate MICs are denoted by white arrows. Plates were analyzed after 48 h of growth at 37 °C. (**c**) Bicarbonate sensitivity of *E. coli* ∆*yqjA* and complementation of bicarbonate sensitivity of *E. coli* ∆*yqjA* by *B. thailandensis dbcA*. Spot assay of 1:10 dilutions of *E. coli* strains (W3110; vec, ∆*yqjA*; vec, ∆*yqjA*; YqjA, and ∆*yqjA*; DbcA) on LB agar media with 50 µg/ml Kan and 20 mM sodium bicarbonate as indicated. (**d**) Bicarbonate sensitivity of *E. coli ∆nhaA*. Spot assay of 1:10 dilutions of indicated strains on LB agar plate with and without 25 mM sodium bicarbonate. (**e**) Effect of bicarbonate on colistin MIC of different bacterial species. MIC was determined for indicated strains on LB agar plates with and without physiological concentration of bicarbonate (~ 25 mM). The pH of growth media was adjusted to pH 7.0 to avoid external pH fluctuations by sodium bicarbonate. Approximate MICs are denoted by white arrows.

It has been reported that sodium bicarbonate can disrupt the PMF by alkalinizing the cytoplasm of *S. aureus*, increasing the susceptibility to polymyxin^39^. Since **∆***dbcA* is alkaline pH sensitive, we hypothesized that it may be sensitive to bicarbonate as well. While 15 mM sodium bicarbonate is not enough to drastically increase the external pH of the MH2 media, **∆***dbcA* was hypersensitive to sodium bicarbonate (Fig. 3a), suggesting that **∆***dbcA* has defective cytoplasmic pH homeostasis. Interestingly, sodium bicarbonate increased the sensitivity of wild type *B. thailandensis* to colistin (Fig. 3b), showing similarity between the effect of alkaline pH and sodium bicarbonate in colistin susceptibility. *E. coli ∆yqjA* is also sensitive to bicarbonate, and this phenotype can be rescued by the overexpression of DbcA (Fig. 3c), supporting the role of DbcA in maintaining cytoplasmic pH homeostasis. NhaA is a Na^+^/H^+^ antiporter and *E. coli ∆nhaA* is also alkaline pH sensitive^48^. *E. coli ∆nhaA* is also sodium bicarbonate sensitive (Fig. 3d), consistent with a previous report^39^, suggesting that bacterial strains unable to maintain cytoplasmic pH homeostasis are also sensitive to sodium bicarbonate, probably due to its cytoplasmic alkalinization effect.

We then measured the colistin MICs for other bacteria in the presence of bicarbonate and observed a similar pattern as alkaline pH. Bicarbonate decreased colistin MIC for a majority of bacteria tested (Fig. 3e). We also observed similar disappearance of heteroresistant colonies of *S. marcescens* with bicarbonate (Fig. 3e). Bicarbonate also increased the colistin sensitivity of heteroresistant *S. marcescens* (see Supplementary Fig. S1 online). Since the pH of the media was buffered to pH 7.0 using 100 mM Tris, we argue that the increased susceptibility to colistin is caused by the alkalinization of cytoplasmic pH, rather than the external pH.

### A link between oxygen and pH homeostasis

**Figure 4:**
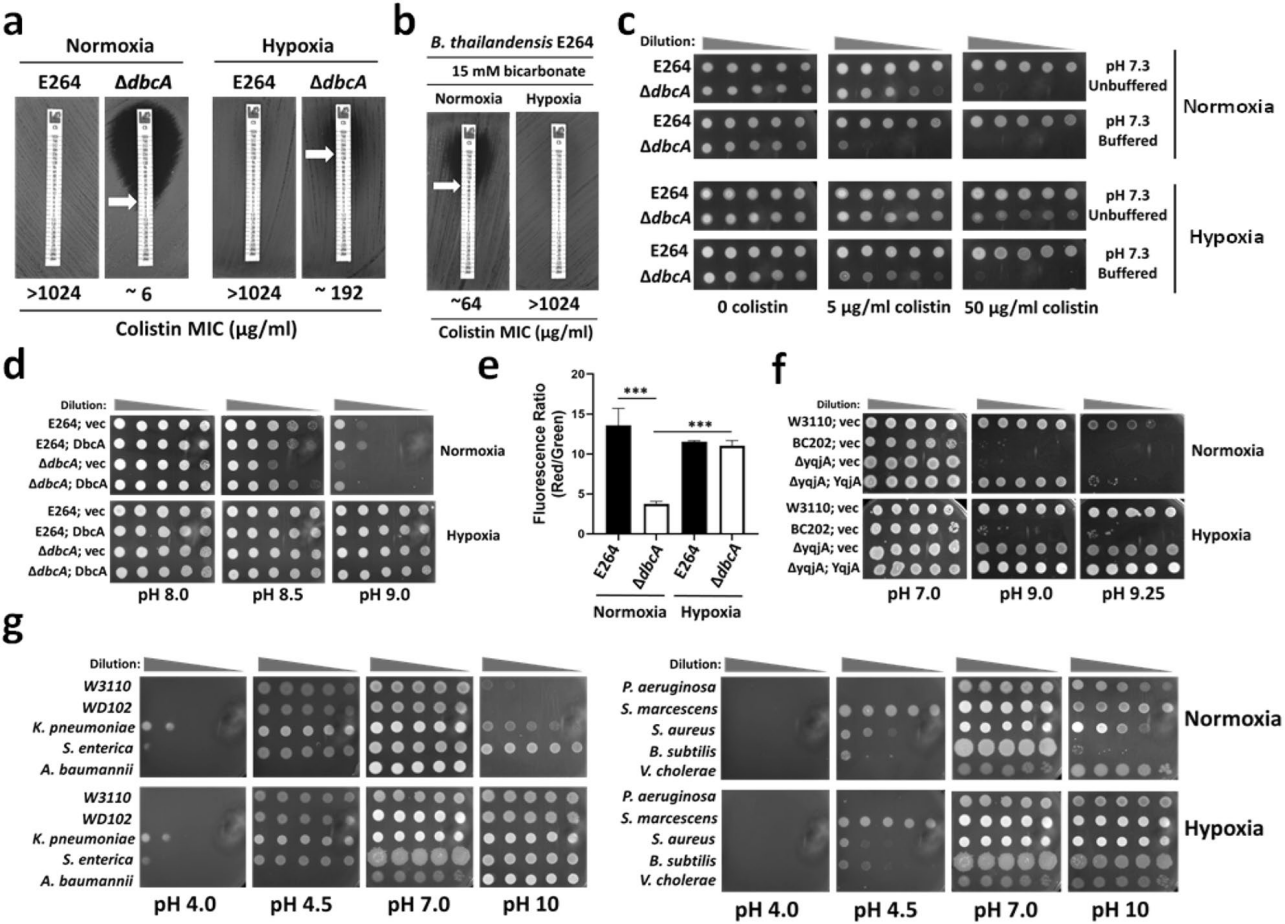
Complementation of alkaline pH sensitivity or colistin sensitivity by hypoxia. (**a**) Partial compensation of colistin sensitivity of *B. thailandensis ∆dbcA* by hypoxia. The colistin MIC was determined for *B. thailandensis* strains (E264 and *∆dbcA*) grown on MH2 agar plates with 100 µg/ml Tmp. (**b**) Reversal of bicarbonate induced sensitization of *B. thailandensis* E264 to colistin by hypoxia. (**c**) The effect of media buffering on colistin resistance. Spot assay of 1:10 dilutions of *B. thailandensis* strains (E264 and *∆dbcA*) were grown in either normoxia or hypoxia on MH2 agar plates supplemented with indicated concentrations of colistin with unbuffered (pH 7.3 adjusted by HCl or NaOH) or buffered media (pH 7.3 adjusted with 70 mM BTP). (**d**) Compensation of alkaline pH stress by hypoxia in *B. thailandensis* strains. Spot assay of 1:10 dilutions of indicated strains on MH1 agar media with 100 µg/ml Tmp, 0.002% rhamnose with different pH media adjusted with HCl or NaOH and grown in either normoxia or hypoxia. (**e**) Compensation of membrane depolarization of *B. thailandensis ∆dbcA* by hypoxia. Membrane potential was measured for *B. thailandensis* strains (E264 and *∆dbcA*) grown in either normoxia or hypoxia. ***; p < 0.001. (**f**) Compensation of alkaline pH stress by hypoxia in *E. coli* strains. Spot assay of 1:10 dilutions of indicated *E. coli* strains at different pH LB media and grown in either normoxia or hypoxia. 70 mM BTP was used to adjust the pH and 50 µg/ml Kan was used in the LB media for plasmid selection. (**g**) Compensation of alkaline pH stress by hypoxia in different bacterial species. Spot assay of 1:10 dilutions of indicated strains at different pH LB agar plates in either normoxia or hypoxia. 100 mM MES was used for pH 4.0 and 5, whereas 70 mM BTP was used for pH 8.0, 9.0. and 10.

∆*dbcA* displayed a more severe growth defect in 25 ml broth when grown in a 250 ml flask compared to when grown in a test tube (Data not shown). We reasoned this could be the effect of aeration or oxygen levels and asked if altering the level of oxygen in growth media also affects the colistin susceptibility of **∆***dbcA*. To answer this, we measured the colistin MIC of *B. thailandensis* E264 and **∆***dbcA* at ambient oxygen levels (normoxia) and low oxygen levels (hypoxia). Surprisingly, we observed that the colistin MIC of **∆***dbcA* increased to ~ 192 µg/ml when grown in hypoxia (Fig. 4a). The reduction of colistin MIC of E264 by bicarbonate could also be compensated by hypoxia (Fig. 4b). The colistin sensitivity of **∆***dbcA* could be further lowered by media buffering and the compensation of colistin sensitivity of **∆***dbcA* by hypoxia could be suppressed by buffering the media (Fig. 4c). *Burkholderia* spp. are known to fluctuate the external pH as they grow, and they are known to acidify the media during stationary phase, regulated by quorum sensing^49^. Using a buffer and restraining the external pH fluctuation by **∆***dbcA* not only further increased its colistin sensitivity, but also suppressed the complementation of colistin sensitivity of **∆***dbcA* by hypoxia (Fig. 4c).

We then investigated whether lowering the level of oxygen could rescue alkaline pH sensitivity of **∆***dbcA*. The alkaline pH sensitivity of **∆***dbcA* was corrected when grown under hypoxia (Fig. 4d). In fact, all strains tested grew better in alkaline pH media under hypoxia compared to normoxia (Fig. 4d). Hypoxia also corrected the lower membrane potential observed in **∆***dbcA* (Fig. 4e). *E. coli* ∆*yqjA* grew much better at pH 9.25 under hypoxia (Fig. 4f). However, BC202^50^ (*E. coli* ∆*yqjA*, ∆*yghB*) could not be rescued at pH 9.25 by hypoxia (Fig. 4f).

It was clear that lowering the amount of oxygen rescues the cells from alkaline stress. To explore this further, we measured the effect of hypoxia at extreme pHs, both alkaline and acidic. We grew several bacterial species at pH’s of 4.0 to 10 under normoxia and hypoxia. We observed that growth under hypoxia rescued all the bacterial strains against extreme alkaline pH whereas their sensitivity to extremely acidic pH stress remained the same under both growth conditions (Fig. 4g), suggesting that only alkaline stress can be alleviated by hypoxia.

In *E. coli*, alkaline stress increases the expression of non-proton pumping cytochrome bd, whereas the expression of proton pumping respiratory chain complexes decrease to minimize proton loss from the cytoplasm when generating PMF^51^. An *E. coli* cytochrome *bd* mutant was also reported to be alkaline pH sensitive^52^. In *E. coli*, the expression of cytochrome *bd* is regulated positively by a transcriptional regulator ArcA under hypoxia^53^. We propose that the induction of non-proton pumping pathways during hypoxia is responsible for rescuing the cells from alkaline pH stress by simply avoiding cytoplasmic proton loss. We reasoned that a *E. coli* cytochrome bd mutant should be sensitive to sodium bicarbonate and may be more sensitive to colistin as well. Indeed, *E. coli* ∆*cydB* was more sensitive to bicarbonate as well as colistin (see Supplementary Fig. S2 online) compared to its parent strain BW25113. This suggests that avoiding cytoplasmic proton loss could rescue cells from colistin toxicity.

### Oxygen dependent colistin resistance in bacteria

**Figure 5:**
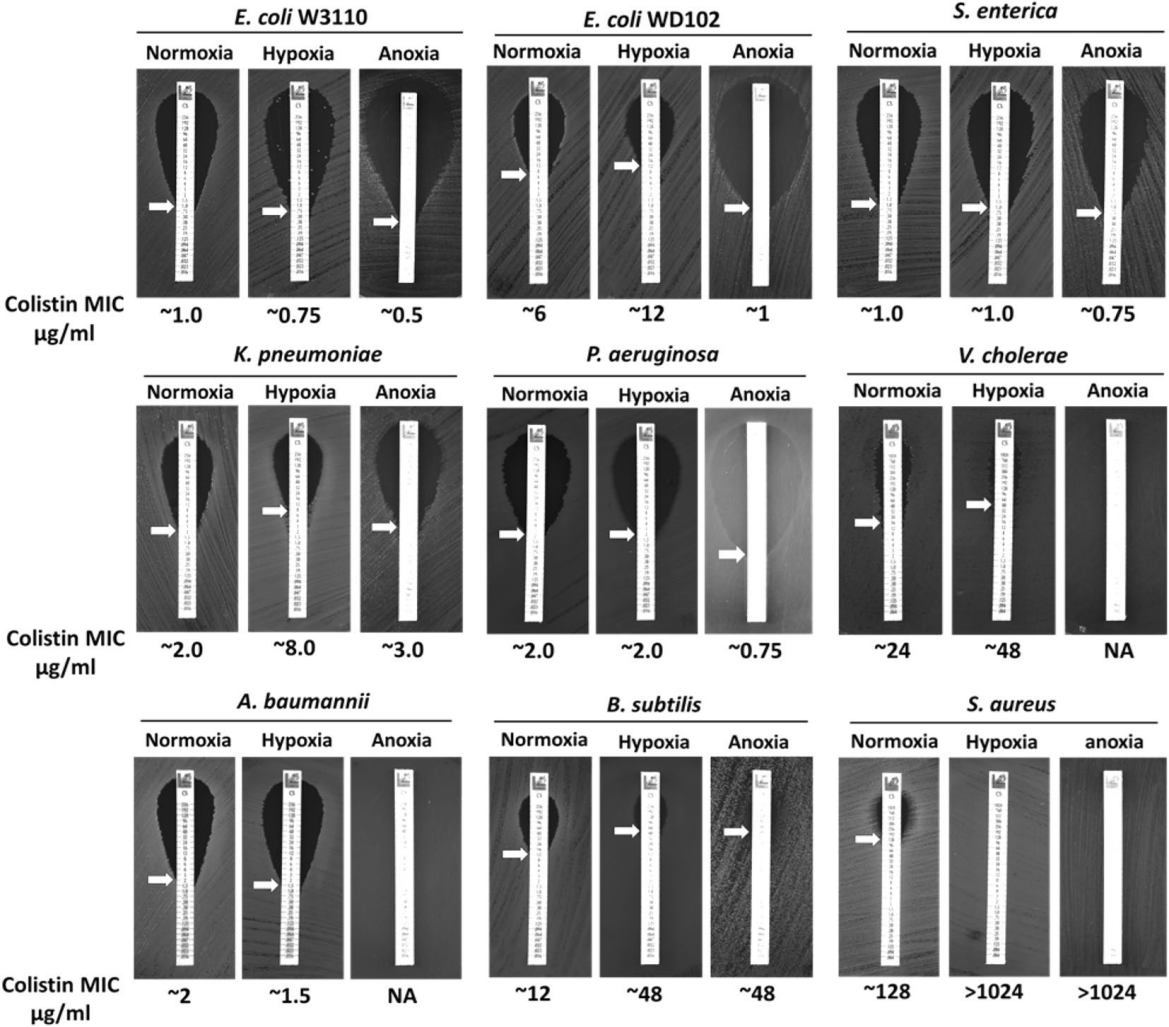
Oxygen dependent colistin resistance in different bacterial species. The colistin MIC was determined for indicated strains on LB agar plates. For anoxia, LB agar plates were supplemented with 20 mM sodium nitrate. Plates were analyzed after 24 h of growth at 37 °C. Approximate MICs are denoted by white arrows.

To examine whether the level of oxygen affects colistin susceptibility, we measured the colistin MIC of different bacteria grown under normoxic, hypoxic, and anoxic conditions. Growth under hypoxia increased the colistin MIC of *E. coli* WD102, *K. pneumoniae*, *Vibrio cholerae*, *S. aureus*, and *Bacillus subtilis* (Fig. 5). However, growth under anoxia decreased the colistin MIC of *E. coli* W3110, *E. coli* WD102, *S. enterica*, and *P*. *aeruginosa*. It should be noted that colistin MIC for several bacteria altered between buffered pH 7.0 LB agar plates (Fig. 2) and unbuffered pH 7.0 LB agar plates (Fig. 5—normoxia plates). *E. coli*, *S. enterica*, *P*. *aeruginosa*, *V. cholerae*, *S. aureus*, and *B. subtilis* displayed different colistin MIC in buffered pH 7.0 LB (Fig. 2) compared to unbuffered pH 7.0 LB (Fig. 5—normoxia plates), further supporting the notion that colistin toxicity is linked to the pH homeostasis.

### Colistin resistance is dependent upon the carbon source

**Figure 6:**
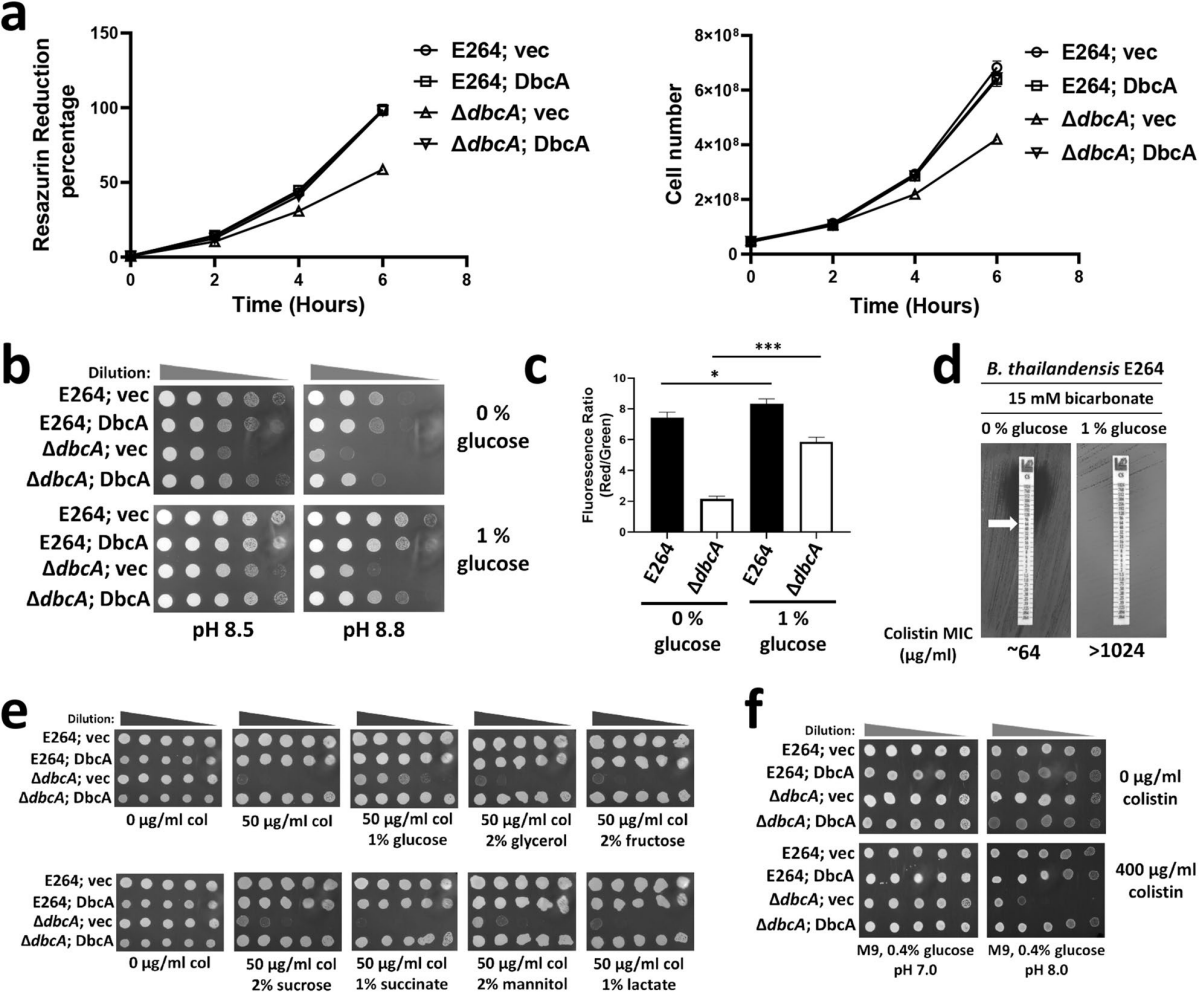
Glucose dependent colistin resistance. (**a**) Lower metabolic activity of *B. thailandensis* ∆*dbcA*. Relative metabolic activity was measured by the percentage of the reduced form of Resazurin within 6 h of growth. 5 × 10^7^ cells of overnight cultures of indicated strains were inoculated in 25 ml fresh MH1 broth, with 100 µg/ml Tmp, 0.015% Resazurin dye and grown for 6 h. Resazurin reduction percentage for each strain was calculated (left) and the cell number was also determined (right) for each time point. (**b**) Compensation of alkaline pH stress by glucose supplementation in the growth media. Spot assay of 1:10 dilutions of all four *B. thailandensis* strains on MH1 media with 100 µg/ml Tmp, 0.002% rha, and 0 and 1% glucose. The pH was adjusted with either HCl or NaOH. (**c**) Partial compensation of membrane depolarization of *B. thailandensis ∆dbcA* by glucose. Membrane potential was measured for *B. thailandensis* strains (E264 and *∆dbcA*) grown in MH2 media with different amounts of glucose as indicated. *; p < 0.05, and ***; p < 0.001. (**d**) Reversal of bicarbonate induced sensitization of *B. thailandensis* E264 to colistin by glucose supplementation. MIC was determined on MH2 agar plates with 15 mM sodium bicarbonate and different concentrations of glucose as indicated. (**e**) Effect of different carbon sources in colistin resistance. Spot assay of 1:10 dilutions of indicated *B. thailandensis* strains in MH2 agar plates with 100 µg/ml Tmp, 0.002% rha, and different carbon sources. Plates were analyzed after 48 h of growth at 37 °C. (**f**) Colistin sensitivity of *B. thailandensis ∆dbcA* in M9 media. 1:10 dilutions of indicated strains were spotted on M9 agar plates with 0.4% glucose with or without 400 µg/ml colistin adjusted to different pH using either HCl or NaOH.

We reported that *B. thailandensis* **∆***dbcA* had lower membrane potential than the parent strain E264^15^. It is possible that **∆***dbcA* also has lower metabolic activity. We used a redox indicator dye, resazurin to measure the metabolic activity of **∆***dbcA*. Active bacterial populations can reduce the resazurin dye from the oxidized form (blue; absorbance at 570 nm) to the reduced form (red; absorbance at 600 nm), giving a quantifiable measure of bacterial metabolic activity^54^. We found that **∆***dbcA* had lower resazurin reduction activity coupled with slower growth at pH 7.5 MH1 media compared to other strains (Fig. 6a). We then supplemented the growth media with glucose as a source of metabolic energy to see if that could rescue the alkaline pH sensitivity of **∆***dbcA*. Addition of glucose could not only partially rescue the alkaline pH sensitivity of **∆***dbcA* (Fig. 6b), but also could partially correct the lower membrane potential of **∆***dbcA* (Fig. 6c). It should be noted that glucose supplementation could also significantly increase the membrane potential of E264 (Fig. 6c) and improve its survivability at extreme alkaline pH (Fig. 6b). Similar to hypoxia, supplementation of glucose also compensated alkaline pH stress of a majority of bacterial species tested (see Supplementary Fig. S3 online). The reduction of colistin MIC of E264 by bicarbonate could also be compensated by glucose supplementation (Fig. 6d), all suggesting that the compensation of glucose supplementation is also linked to the pH.

We then tested whether supplementation of different carbon sources (glucose, glycerol, fructose, sucrose, succinate, mannitol, lactate, acetate, and citrate) could compensate the colistin sensitivity of **∆***dbcA.* We found that while supplementation of glucose slightly compensates the colistin sensitivity of **∆***dbcA* in MH2 agar media (Fig. 6e), acetate and citrate reduced the colistin MIC of **∆***dbcA* (see Supplementary Fig. S4 online). In fact, **∆***dbcA* was slightly more sensitive to acetate and citrate compared to the WT E264 (see Supplementary Fig. S4 online). Surprisingly, the colistin MIC of **∆***dbcA* in M9 agar media with 0.4% glucose was more than 1024 µg/ml, similar to WT E264 (see Supplementary Fig. S4 online). This is consistent with a previous finding, where susceptibility of several bacterial species to polymyxin was increased in rich medium compared to minimal medium^55^. During the growth of *B. thailandensis* in rich media, such as LB, the pH of the supernatant increases initially and later decreases during the late stationary phase^49^. It has been proposed that the deamination of amino acids in LB media leads to the production of ammonia, that is responsible for medium alkalinization^49^. We propose that the medium alkalinization in rich media is responsible for the colistin hypersensitivity of **∆***dbcA.*

**Figure 7:**
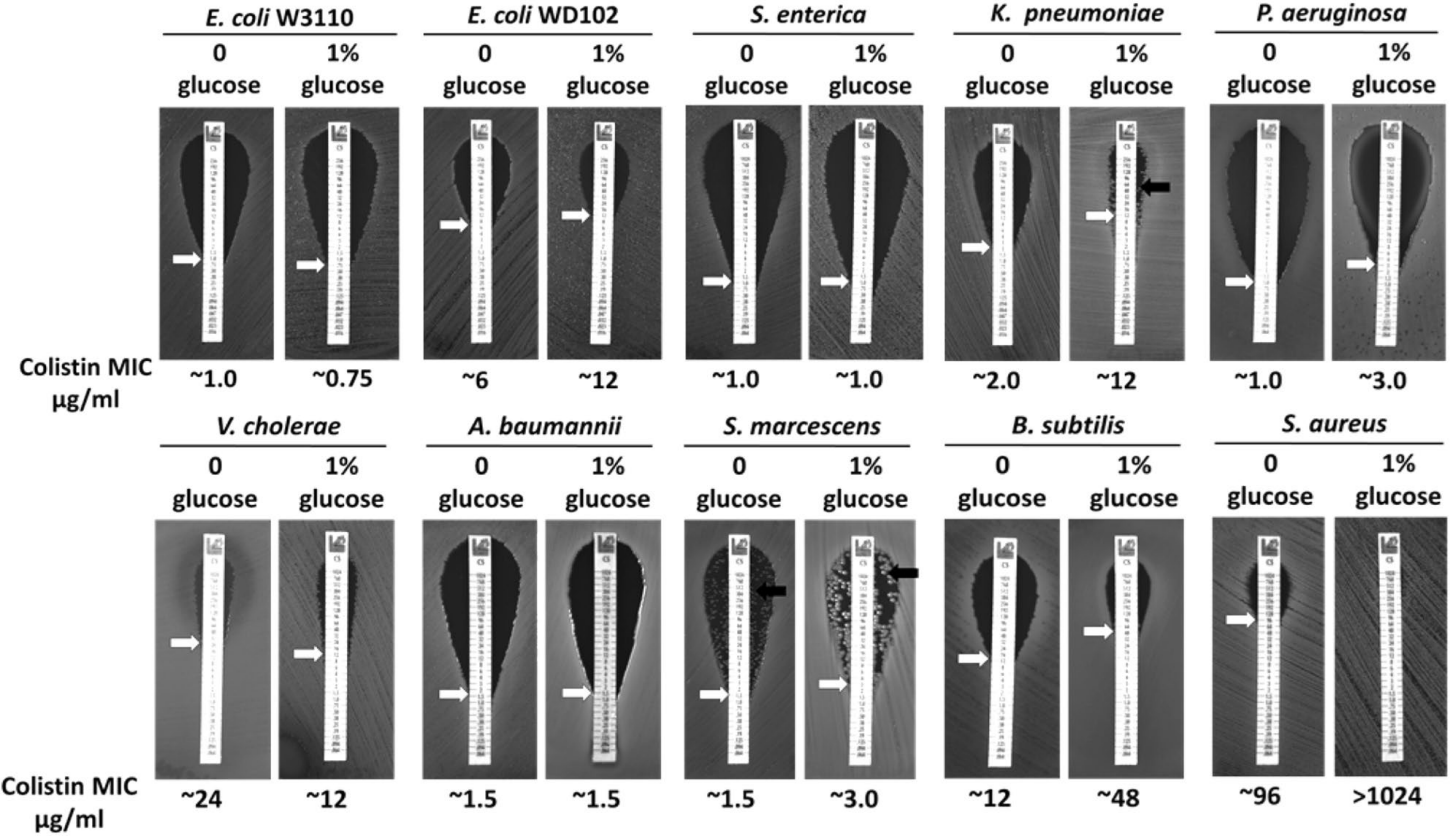
Effect of glucose supplementation in colistin MIC of different bacterial species. The colistin MIC was determined for indicated strains on LB agar plates with either 0% or 1% glucose. Plates were analyzed after 24 h of growth at 37 °C. Approximate MICs are denoted by white arrows. A black arrow represents heteroresistant colonies of *S. marcescens or K. pneumoniae*.

When the initial pH of the M9 media with 0.4% glucose is adjusted to pH 8.0, **∆***dbcA* was much more sensitive to colistin (Fig. 6f), supporting our hypothesis that the medium alkalinization is sensitizing **∆***dbcA* to colistin. We also measured the external pH fluctuation by E264 and **∆***dbcA* with different sugars. We found that with glucose, the pH of the supernatant dropped compared to other sugars (see Supplementary Fig. S5 online). We then measured the colistin MIC of different bacterial species with glucose supplementation. We found that addition of glucose increased the colistin MIC for *E. coli* WD102, *K. pneumoniae*, *P. aeruginosa*, *S. marcescens*, *B. subtilis*, and *S. aureus* (Fig. 7) all supporting a general link between cytoplasmic pH and colistin sensitivity. Our observation is consistent with previous finding regarding the effect of glucose in polymyxin resistance of *P. aeruginosa*^56^.

## Discussion

**Figure 8:**
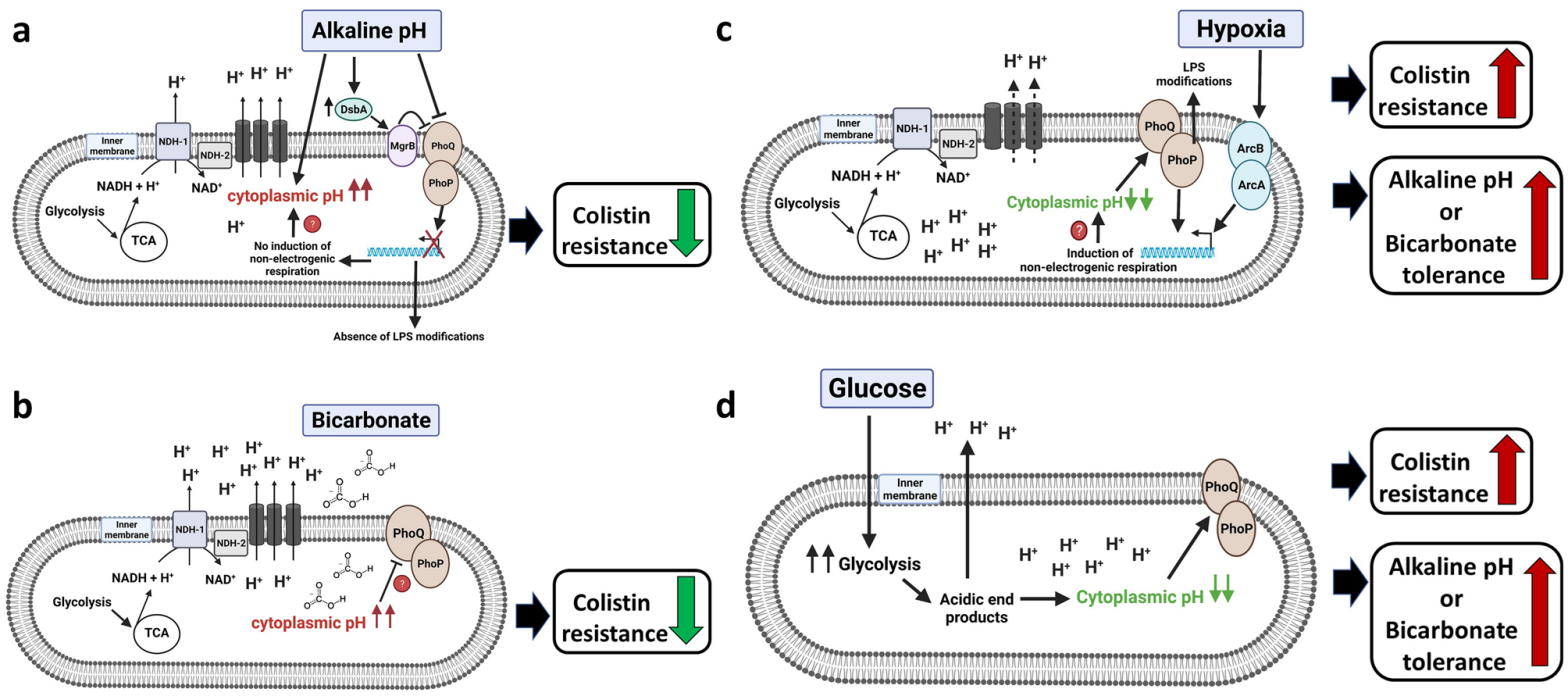
Model depicting the link between cytoplasmic pH and colistin resistance. Only the bacterial cytoplasmic membrane is depicted for simplicity. (**a**) While *E. coli* maintains its cytoplasmic pH at ~ 7.2 when grown at pH 7.0, growth at pH 8.0 can increase the cytoplasmic pH to ~ 7.6^88^. Increasing cytoplasmic pH can be toxic to cells unless compensated by cytoplasmic pH homeostasis mechanisms, one of which includes induction of non-proton pumping respiratory pathways^51^. Polymyxin has been shown to inhibit the non-proton pumping respiratory complex NDH-2^59, 60^. Growth at alkaline pH in minimal media inhibits the expression of PhoPQ dependent genes^69, 70^. DsbA is a periplasmic disulfide-bond formation protein, which is induced by alkaline pH^80, 89^. MgrB is a small inner membrane peptide that directly binds PhoQ and mediates negative feedback on the PhoPQ circuit^36^. DsbA has been reported to negatively regulate PhoPQ probably through disulfide bond formation in MgrB^35^. Inhibition of PhoPQ can reduce colistin resistance either through inhibition of LPS modifications or through inhibition of non-proton pumping respiratory pathways^33^, which could further increase the cytoplasmic pH and increase colistin toxicity. (**b**) Sodium bicarbonate has been reported to increase the cytoplasmic pH of *S. aureus*^39^. This increase in cytoplasmic pH may be responsible for reduced colistin resistance. The cytoplasmic domain of PhoQ is responsible for sensing acidic cytoplasmic pH and activating PhoP in *S. Typhimurium*^90^. Therefore, it is possible that bicarbonate may repress PhoPQ by causing an alkaline cytoplasmic pH leading to sensitivity to colistin. (**c**) Growth in hypoxia stimulates ArcAB, which can induce non-proton pumping respiratory complex such as cytochrome bd^53^. This could reduce cytoplasmic proton loss and decrease the cytoplasmic pH, increasing alkaline pH tolerance. Cytoplasmic acidification could further activate PhoPQ and increase colistin resistance. (**d**) Glucose supplementation of *B. thailandensis* can acidify the growth media, which may explain the similar increase in alkaline pH tolerance as hypoxia. This hypothesis is supported by the observed cytoplasmic acidification by glucose supplementation in *Bacillus pseudofirmus*^86^. The cytoplasmic acidification by glucose could also stimulate PhoPQ.

There is emerging evidence regarding the link between cytoplasmic pH and antibiotic resistance. In *E. coli*, cytoplasmic alkalinization by chloroquine was shown to decrease norfloxacin resistance^57^. Chloroquine can also decrease colistin MIC of both *E. coli* W3110 and *E. coli* WD102 (see Supplementary Fig. S6 online). It has been reported that intracellular alkalinization caused by kanamycin, chlorpromazine, and norfloxacin can cause cell death in *Mycobacterium smegmatis*, while increasing proton influx by either media acidification or by using protonophore can block the antibiotic lethality^58^. Our study supports this emerging hypothesis and suggests that colistin resistance of a number of bacterial species can be attenuated by intracellular alkalinization by either alkali challenge or adding bicarbonate. Similarly, colistin sensitivity can be rescued by growing cells under conditions, such as acidic pH, hypoxia, or glucose supplementation, all of which could reduce cytoplasmic alkalinization. This is the first report to our knowledge that shows a link between pH, oxygen and colistin resistance in bacteria. The findings of this study are depicted Fig. 8.

Why alkaline pH stress/bicarbonate decreases colistin resistance remains an important question. It has been reported that polymyxin is a non-competitive inhibitor of the type II NADH-quinone oxidoreductase (NDH-2) respiratory enzyme in *E. coli*, *Acinetobacter baumannii*, *K*. *pneumoniae*^59^, and *M*. *smegmatis*^60^. NDH-2 is an alternative NADH quinone reductase, which oxidizes NADH coupled to the reduction of quinone without pumping protons across the inner membrane^61^. It is possible that upon inhibition of non-proton pumping pathways by polymyxin, cells rely solely on proton pumping pathways that would be devastating under alkaline pH condition, unless compensated by increasing proton influx or switching to hypoxic metabolism or fermentation, which avoid intracellular alkalinization. The polymyxin B-induced inhibition of NDH-2 and the metabolic shift from aerobic to fermentation in *K. pneumoniae* supports this hypothesis^62^. However not all bacteria have NDH-2, including *B. thailandensis*^63, 64^. The reduced colistin MIC of *E. coli* ∆*cydB* (see Supplementary Fig. S2 online) also suggests that induction of non-proton pumping cytochrome bd can increase colistin resistance. Whether polymyxin also inhibits the non-proton pumping pathway at cytochrome bd level, or ubiquinone level, is unknown.

Our data suggests that the pH, bicarbonate, oxygen, and glucose dependent colistin toxicity is observed mainly for colistin resistant Gram-negative bacterial species such as *E. coli* WD102, *K. pneumoniae*, *V. cholerae*, *B. thailandensis* and *S. marcescens* as well as Gram-positive bacterial species including *S. aureus* and *B. subtilis*. All these colistin resistant Gram-negative species have a conserved PhoPQ or PmrAB systems^65–68^. It is possible that alteration of lipid A modifications at alkaline pH is responsible for reduction of colistin MIC. It has been reported that PhoPQ-dependent gene expression increases upon decreasing the pH of the media in *S. typhimurium*^69^ and *S. marcescens*^68^. In *S. typhimurium*, lipid A modifications with Ara4N and phosphoethanolamine (PEtN) were suppressed at pH 8.0 N-minimal media with 10 mM Mg^2+^ not only in the WT strain, but also in the *PhoP*^c^ and *pmrA*^c^ mutants^70^. However, it should be noted that these experiments in *S. typhimurium* and *S. marcescens* were performed using minimal media. While *Salmonella* PhoP-dependent gene expressions in murine RAW264.7 macrophages has been correlated with acidification of the phagosome, acidification of LB broth in vitro did not show a similar level of PhoP-dependent gene expression^71^. Our experiment also shows that the growth of *S. typhimurium* at different pH LB media only showed a slight decrease in colistin MIC from 0.5 µg/ml at pH 5.5 to 0.3 µg/ml at pH 8.0 (Fig. 2). Therefore, measuring the expression of PhoPQ or PmrAB-dependent genes at different pH in LB media will help explain whether the decrease in colistin MIC with alkali challenge is due to the reduced expression of PhoPQ or PmrAB systems. The decrease in colistin MIC of *B. cenocepacia* MH55 (ArnT suppressor mutant strain)^10^ and *K. pneumoniae arnT*::Tn^72^ (see Supplementary Fig. S7 online) by alkaline pH supports lipid A modification independent colistin resistance. Our previous report shows that *B. thailandensis* E264 has similar level of Ara4N modified lipid A species grown at different pH media (pH 5.5, 7.0, and 7.5)^16^. However, E264 is more sensitive at pH 7.5 media compared to pH 7.0 (see Supplementary Fig. S7 online). Whether suppression of lipid A modifications at alkaline pH is responsible for reduced colistin MIC in other Gram-negative bacterial species remains to be investigated. Quantitative measurement of modified lipid A species of different bacterial species at different pH LB media will help address this hypothesis.

Our findings suggest that the external pH effect on colistin MIC can be independent of lipid A modification with Ara4N. However, it is also possible that alkaline pH could suppress other LPS modifications beside Ara4N addition by ArnT that could be responsible for pH dependent colistin resistance. In addition, with intact PhoPQ or PmrAB systems, it is possible that the pH dependent colistin resistance is mediated directly or indirectly through PhoPQ or PmrAB, independent of lipid A modifications. The PhoPQ system has been shown to regulate many processes including cytoplasmic pH regulation such as induction of non-proton pumping pathways^33^ that could prevent cytoplasmic proton loss and increase colistin resistance (Fig. 8a). Whether bicarbonate also inhibits PhoPQ by cytoplasmic alkalinization remains to be investigated (Fig. 8b). The reduction of colistin MIC in Gram-positive bacteria, such as *B. subtilis* and *S. aureus* at alkaline pH (Fig. 2) is also not clear. Similar to LPS modifications by Gram-negative bacteria, Gram-positive bacteria can also alter their cell surface charge either by modifying their negatively charged cell wall teichoic acids with positively charged _D_-alanine mediated by *dltABCD* operon or by masking their anionic membrane phosphatidylglycerols with cationic _L_-lysine mediated by the enzyme MprF^73^. Interestingly, it has been reported that growth at alkaline pH significantly reduced the alanine ester content of teichoic acids only in chemostat culture of *S. aureus*, probably due to their removal by the hydrolysis of the base-labile ester linkages at high pH^74, 75^. This may explain the reduced colistin MIC of *S. aureus* and *B. subtilis* at alkaline pH. The enzyme MprF and *dltABCD* operons are regulated by GraXSR three component system^76^ and whether GraXSR can sense the change in pH remains to be investigated.

Reduction in membrane potential at alkaline pH may explain the reduced colistin MIC. The lower membrane potential observed in ∆*dbcA* at pH 7.5 was corrected by either acidic pH^16^, or hypoxia (Fig. 4e), or supplementation of glucose (Fig. 6c), paralleled with increase in colistin resistance (Figs. 4a, 6e). The increased sensitivity towards colistin due to lower membrane potential is also supported by other reports that show reducing membrane potential by CCCP can sensitize many bacterial species to colistin^15, 32^. It is also possible that PMF dependent secondary transporters required for colistin efflux are inefficient at alkaline pH due to a reduced proton gradient and lower PMF^51, 52^. However, addition of sodium bicarbonate has been shown to increase the cytoplasmic pH of *S. aureus* with a subsequent increase in ∆ѱ, and it is this increase in ∆ѱ (membrane hyperpolarization) that has been proposed to be responsible for increased colistin sensitivity^39^. This is further supported by *S. aureus* ∆*atpA* and *E. coli* ∆*phoP*, both of which show membrane hyperpolarization and increased sensitivity towards polymyxin^33, 34^.

We propose that a pH effect, rather than membrane potential, could explain colistin resistance more accurately. It has been proposed that the bactericidal activity of polymyxin requires the respiratory generation of PMF in *E. coli* and membrane depolarization by CCCP exposure was shown to improve survival against colistin^33^. It should be noted that the aerobic generation of PMF could increase the cytoplasmic alkalinization over time and could be worse at alkaline pH unless compensated by proton influx. Brief exposure to CCCP could in fact acidify the cytoplasm^58^ and avoid cytoplasmic alkalinization to a toxic level, thus improving colistin resistance. However, the continuous presence of CCCP in the growth media sensitizes many bacterial species to colistin probably due to lower metabolic activity and inhibition of PMF dependent transporters required for colistin resistance^15, 32^. The compensation of reduction of colistin MIC of E264 by CCCP by either acidic pH, glucose supplementation, or hypoxia supports the notion that the CCCP effect is also linked to pH homeostasis (see Supplementary Fig. S8 online). The decrease in colistin resistance of *S. aureus* by bicarbonate may also be due to increase in cytoplasmic pH to a toxic level^39^. However, the inhibition of aerobic respiration by bicarbonate could also be responsible^39^. It should be noted that polymyxin itself could inhibit oxygen consumption in *E. coli*^77^ and *S. typhimurium*^78^. There is evidence that alteration of oxygen consumption is responsible for either bactericidal or bacteriostatic effect of antibiotics in bacteria^79^. It is possible that alkaline pH or bicarbonate affects oxygen consumption and could be responsible for increased sensitivity towards colistin, which may itself inhibit oxygen consumption. Measuring oxygen consumption rates at different pH media or with bicarbonate supplementation should provide more insights regarding the above hypothesis.

It may be argued that increased oxidative stress at alkaline pH may be responsible for increased colistin susceptibility. However, oxidative stress-induced genes are activated at acidic pH in *E. coli* K-12 rather than alkaline^80^. Polymyxin has also been reported to induce oxidative stress^40–44^. Although the increase in colistin MIC of *B. thailandensis* ∆*dbcA*, *E. coli* WD102, *K. pneumoniae*, *V. cholerae*, *B. subtilis* and *S. aureus* under hypoxia supports this notion, reduced colistin MIC of *E. coli* WD102, *K. pneumoniae*, and *P. aeruginosa* in anoxia do not support the oxidative stress hypothesis (Fig. 5). Increase in colistin susceptibility in anaerobic conditions in *P. aeruginosa* biofilms has also been reported^81^, consistent with our own data that shows reduced colistin MIC of *P. aeruginosa* in anoxia, hence questioning the validity of oxidative stress hypothesis. Surprisingly, the exposure to oxidative stress (3.5 mM H_2_O_2_) increased the colistin MIC of *P. aeruginosa* PAO1 by 2–fourfold^82^. Therefore, we argue that a small decrease in colistin MIC of *P. aeruginosa* PAO1 in alkaline pH (Fig. 2) is probably not due to oxidative stress. Here, we propose an alternative explanation. Induction of non-proton pumping pathway under hypoxia may help bacteria avoid increase in cytoplasmic pH to a toxic level, thus compensating extreme alkaline pH as well as colistin sensitivity (Figs. 4, 5). However, in anoxic condition, generation of PMF by nitrate reduction coupled with continuous proton pumping may increase the possibility of losing cytoplasmic protons and increase sensitivity towards colistin.

Carbon starvation during growth under alkaline pH media may be responsible for alkaline pH or colistin sensitivity. *E. coli* XylE is a PMF dependent sugar transporter belonging to MFS superfamily and sharing 63% sequence similarity with human GLUT1^83^. In *B. subtilis*, polymyxin B decreases intracellular levels of carbon metabolites and ATP, while addition of glucose increases survival against polymyxin B by increasing ATP levels^84^. A metabolomics study of colistin treated *Mycobacterium tuberculosis* suggests that colistin induces a shift towards glucose utilization for energy and upregulation of glyoxylate cycle^85^. It is possible that the lower PMF at alkaline pH negatively affects PMF dependent sugar transporters such as *E. coli* XylE and cause sugar starvation, which could be exacerbated by colistin. Our data shows that supplementation of glucose improves bacterial survival against alkaline pH stress or colistin (Fig. 7 and see Supplementary Fig. S3 online), supporting the above hypothesis. We also measured the colistin sensitivity of ∆*dbcA* with other carbon sources and found that the colistin sensitivity compensation was only seen with fermentable carbon source-glucose (see Supplementary Fig. S6 online). The acidification of the media with glucose supplementation (see Supplementary Fig. S5 online) suggests that the glucose effect is also linked to the pH. We propose that glucose could decrease the internal pH by making acidic end products. During a pH shift from 8.5 to 10.5, alkaliphilic bacteria *Bacillus pseudofirmus* OF4 displayed cytoplasmic pH of 9.2 in the presence of glucose instead of pH 10.5 in the presence of malate^86^ supporting our hypothesis that supplementation of glucose could acidify the cytoplasm or avoid cytoplasmic alkalinization and hence improve survival against colistin. The reduction of colistin MIC of ∆*dbcA* by acetate or citrate (see Supplementary Fig. S4 online) might also be linked to the cytoplasmic pH homeostasis. Acetate and citrate could directly feed into Krebs’s cycle and increase electron transport chain (ETC) activity, hence pumping out cytoplasmic protons by ETC complexes and increasing the possibility of cytoplasmic alkalinization to a toxic level.

How DbcA maintain cytoplasmic pH homeostasis and extreme colistin resistance of *B. thailandensis* is still unclear. Structural and biochemical studies of this interesting family of membrane transporters will help answer these questions. Future investigations including direct measurement of cytoplasmic pH under different growth conditions will further validate our model (Fig. 8) and enhance our understanding of alkaline pH homeostasis and polymyxin resistance. Our work shows how different factors such as pH, level of oxygen, and source of carbon in the growth media can influence colistin resistance of several bacterial species and hence emphasizes a need to take caution while measuring colistin MIC in a laboratory setting.

## Materials and methods

### Culture conditions

The bacterial strains and plasmids used in this study are listed in Table 1. Bacteria were grown in either LB medium (1% tryptone, 0.5% yeast extract, and 1% NaCl), Mueller Hinton Broth (MH1), Cation-adjusted Mueller Hinton Broth 2 (MH2) (Sigma). MH1 or MH2 as indicated was used exclusively with *B. thailandensis* and LB was used for all other strains. Colistin (Col), trimethoprim (Tmp), rhamnose (rha), 2,6 Diaminopimelic acid (DAP), kanamycin (kan), Resazurin, sodium bicarbonate, glucose, glycerol, fructose, sucrose, mannitol, sodium lactate, sodium acetate, chloroquine diphosphate salt, and sodium citrate were purchased from VWR or MilliporeSigma. Cultures were grown at 37 °C shaking at 225 rpm. BD GasPak EZ Gas Generating Container Systems with either microaerophilic or anaerobe sachets were purchased from VWR. Colistin MIC strips were purchased from Liofilchem, Inc.

**Table 1:** Bacterial strains and plasmids used in this study.

Strains	Description	Source or reference
*Escherichia coli* W3110	Wild Type, F^−^, λ^−^, IN (*rrnD-rrnE)1*, *rph-1*	*E. coli* genetic stock center, Yale University
*Escherichia coli* W3110; vec	W3110 transformed with pBBR1MCS-2	^15^
*Escherichia coli* ∆*nhaA*	W3110, ∆*nhaA*-737::kan	^22^
*Escherichia coli* BW25113	Wild Type, lacI^q^ *rrnB*_T14_ ∆*lacZ*_WJ16_ *hsdR514* ∆*araBAD*_AH33_ ∆*rhaBAD*_LD78_	^91^
*Escherichia coli* BW25113 *∆cydB*	Kanamycin-resistant Keio collection mutant	^92^
*Escherichia coli* BC202KS	W3110 ∆*yqjA*::Tet^R^ ∆*yghB781::*Kan^S^	^15^
*Escherichia coli* BC202; vec	BC202KS transformed with pBBR1MCS-2	This study
*Escherichia coli* ∆*yqjA*	W3110; ∆*yqjA*::Tet^R^	^50^
*Escherichia coli* ∆*yqjA*; vec	∆*yqjA* transformed with pBBR1MCS-2	This study
*Escherichia coli* ∆*yqjA*; YqjA	∆*yqjA* transformed with pRP102	This study
*Escherichia coli* ∆*yqjA*; DbcA	∆*yqjA* transformed with pRP101	This study
*Escherichia coli* RHO3	SM10(lambda *pir*), kan^S^; ∆*asd::FRT* ∆*aphA::FRT*	^93^
*Burkholderia thailandensis* E264; vec	E264 transformed with pSCrhaB2 Tmp^R^	^15^
*Burkholderia thailandensis* E264 Δ*dbcA*; vec	Δ*dbcA*::FRT transformed with pSCrhaB2 Tmp^R^	^15^
*Burkholderia thailandensis* E264; DbcA	E264 transformed with pSC*dbcA*	^15^
*Burkholderia thailandensis* E264 Δ*dbcA*; DbcA	Δ*dbcA*::FRT transformed with pSC*dbcA*	^15^
*Escherichia coli* WD102	W3110, *pmrA*^*C*^, *zjd-2211*: Tn10	^94^
*Klebsiella pneumoniae* ST258	A clinical isolate from Public Health England resistant to polymyxin	^19^
*Klebsiella pneumoniae arnT*::Tn	A transposon insertion mutant carrying a chloramphenicol resistance determinant (T30)	^72^
*Salmonella enterica* subsp. *Enterica* 14028	Virulent wild type strain	ATCC
*Pseudomonas aeruginosa* PAO1	Wild type strain	^95, 96^
*Vibrio cholerae* C6706	O1 El Tor strain	^97^
*Serratia marcescens* 13880	Wild type strain	ATCC
*Staphylococcus aureus* subsp. *aureus* Rosenbach, 6538	Wild type strain	ATCC
*Bacillus subtilis* 6051	Wild type strain	ATCC
*Burkholderia cenocepacia* K56-2	ET12 clone related to J2315, CF clinical isolate	BCRRC, *B. cepacia* Research and Referral Repository for Canadian CF Clinics
*Burkholderia cenocepacia* MH55	K56-2, Δ*arnT-arnBC*^+^ *lptG*_D31H_ (*lptG*^S^)	^98^
**Plasmids**		
pBBR1MCS-2	Expression vector; RK2 ori, lacZa, T3 and T7 promoters, Kan^R^	^99^
pRP101	pBBR1MCS-2 expressing *dbcA*	This study
pRP102	pBBR1MCS-2 expressing Ec*yqjA*	This study
pSCrhaB2	Expression vector; ori_pBBR1_*rhaR*, *rhaS*, *P*_rhaB_Tmp^R^*mob* +	^100^
pSC*dbcA*	pSCrhaB2 expressing *dbcA* with His_6_ tag at C terminus	^15^

### Transformation and complementation analysis

Transformation of *E. coli* was carried out using a heat shock^87^, while transformation of *B. thailandensis* was carried out using biparental conjugation^16^. Briefly, both recipient *B. thailandensis* and donor *E. coli* RHO3 strains carrying Tmp^R^ plasmid(s) to be transferred were inoculated by thoroughly spreading them on LB plates supplemented with 200 µg/ml DAP and incubated at 37 °C. After ~ 18 h of incubation, a loopful of bacterial cells from conjugation plates were streaked on LB with 100 µg/ml Tmp plates for selection and DAP was excluded on these plates to select against the donor strain, RHO3. After 48 h of incubation at 37 °C, isolated Tmp^R^ colonies were used for colony PCR using plasmid specific primers^16^ to confirm the introduction of Tmp^R^ plasmids into *Burkholderia* recipient strains.

### Susceptibility assays

For testing the susceptibility on solid medium, overnight cultures of bacterial strains were adjusted to 3 × 10^8^ cells/ml. Five microliters of serially log_10_-diluted cells was then spotted on LB/MH1/MH2 agar plates containing various concentrations of antibiotics, sodium bicarbonate, or other chemical agents as indicated in figure captions. Growth was analyzed after incubation for 24–72 h at 37 °C as indicated. The colistin MIC was measured using Liofilchem® MIC Test Strips. Overnight cultures were adjusted to 1 × 10^8^ cells/ml for *B. thailandensis* strains and 5 × 10^7^ cells/ml for all other strains in fresh LB/MH2 and a sterile swab was used to create a lawn of cells. Then the MIC strip was applied to the plates and the growth was evaluated after 24–48 h at 37 °C. All experiments were repeated at least three times and highly reproducible.

### Measurement of membrane potential

Measurement of membrane potential was performed using JC-1 dye^15^. Briefly, 5 × 10^7^ cells from the overnight cultures were inoculated in 25 ml of fresh MH2 broth in 250 ml flask and grown for about 5 h at 37 °C shaking incubator with glucose if required. For hypoxic growth, a bacterial culture was inoculated in 10 ml MH2 broth in 50 ml flask that was placed in BD GasPak EZ Gas Generating Container Systems with microaerophilic sachets (VWR) and grown for 5 h at 37 °C with shaking. Similarly, a 50 ml flask with a 10 ml bacterial inoculation was grown at normal oxygen condition for normoxia control. Then, ~ 6 × 10^8^ cells were taken from each culture, washed with permeabilization buffer, PB (10 mM Tris, pH 7.5, 1 mM EDTA, 10 mM glucose) and, finally, resuspended in PB buffer. 3 µM of JC-1 dye was added, incubated in the dark at 37 °C for 30 min. Cells were washed and resuspended in PB buffer and fluorescence measurements were carried out using a JASCO FP-6300 spectrofluorometer. Membrane potential is determined by the ratio of red (595 nm) to green (530 nm) fluorescence with excitation of 488 nm.

### Measurement of metabolic activity

The metabolic activity was measured using a commercially available resazurin dye (VWR). Measuring cell-viability by resazurin protocol by Tip Biosystems was used to quantitatively measure the resazurin reduction percentage. 5 × 10^7^ cells of overnight bacterial cultures were inoculated in 25 ml fresh MH1 broth, with 100 µg/ml Tmp, and 0.015% resazurin dye and grown at 37 °C shaking incubator for 6 h. Initial absorbance of resazurin at 570 nm (reduced form) and 600 nm (oxidized form) was reported before starting the incubation. After every hour, one ml of culture was removed, centrifuged, and the supernatant was used to measure absorbance at 570 and 600 nm. Pelleted cells were resuspended in PBS and cell number was measured for the same time points.

### Statistical analysis

Experiments were repeated three times with three biological replicates. The data presented in the graphs indicate the mean ± standard deviation (SD) value for three independent replicates of each treatment. GraphPad Prism 9.0 was used to produce graphs and calculate the statistical significances by unpaired Student’s *t*-test.

## Supplementary Information

Supplementary Figures.
