# Supplementary material for: A link between pH homeostasis and colistin resistance in bacteria
Source: Sci Rep. 2021 Jun 24;11:13230. doi: 10.1038/s41598-021-92718-7 (PMC8225787; doi:10.1038/s41598-021-92718-7)
Supplement: Supplementary file 1 — Supplementary Figures. [file 41598_2021_92718_MOESM1_ESM.pdf]

Supplementary Materials for:

A link between pH homeostasis and colistin resistance in bacteria

Pradip R. Panta and William T. Doerrler*

Department of Biological Sciences, Louisiana State University, Baton Rouge, LA, USA

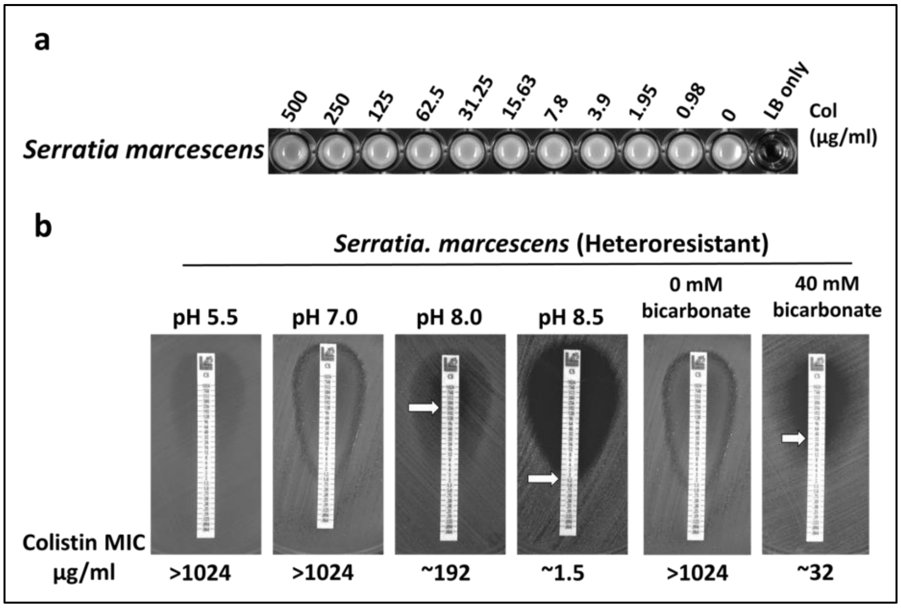

Supplementary Figure S1. Effect of pH or bicarbonate on colistin MIC of *Serratia marcescens* and a heteroresistant clone. **a)** Colistin MIC of *S. marcescens* using broth microdilution method. The 96 well plate was grown at 37°C shaking and analyzed after 24 hours. **b)** Reduction of extreme colistin resistance of heteroresistant *S. marcescens* by alkaline pH or bicarbonate. Colistin heteroresistant colonies of *S. marcescens* were restreaked on 1000 µg/ml colistin LB plates and used for measuring MIC on LB agar media adjusted to different pH or with 40 mM bicarbonate. 100 mM MES was used for pH 5.5, whereas 100 mM Tris was used for all other plates. Approximate MICs are denoted by white arrows.

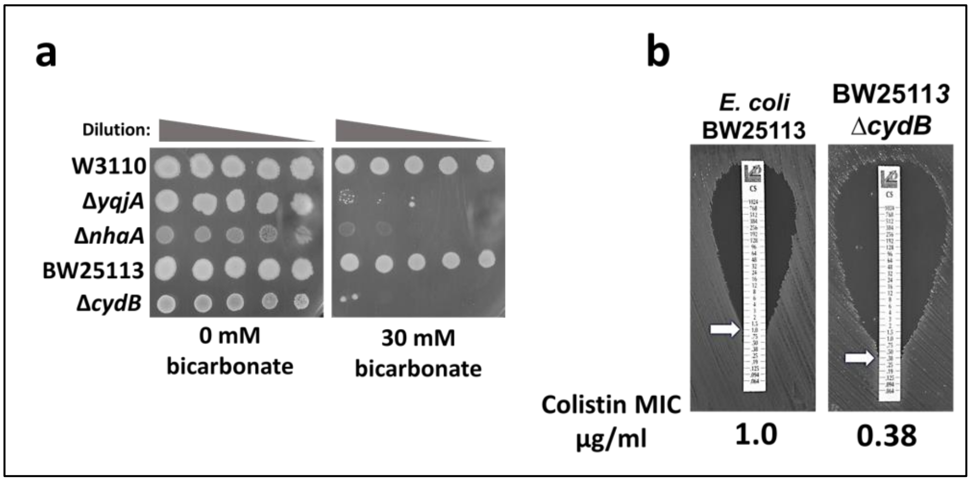

Supplementary Figure S2. Bicarbonate and colistin sensitivity of *E. coli* Δ*cydB*. **(a)** Bicarbonate sensitivity of *E. coli* Δ*cydB*. Spot assay of 1:10 dilutions of indicated strains was carried out on LB agar plates with and without 30 mM sodium bicarbonate. The initial pH of the media was adjusted to pH 7.0 using HCl to avoid the effect of alkalinization of the media by sodium bicarbonate. **(b)** Colistin MIC of *E. coli* BW25113 and Δ*cydB*. Approximate MICs are denoted by white arrows.

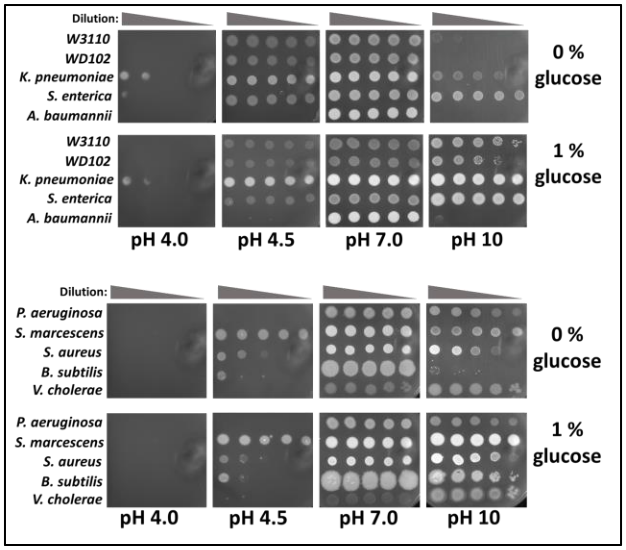

Supplementary Figure S3. Compensation of extreme alkaline pH stress with glucose supplementation. Spot assay of 1:10 dilutions of indicated strains on LB agar adjusted to different pH and supplemented with 1% glucose as indicated. 100 mM MES was used for pH 4.0 and pH 4.5. 70 mM BTP was used for pH 7.0 and pH 10. Plates were analyzed after 36 hours of growth at 37°C.

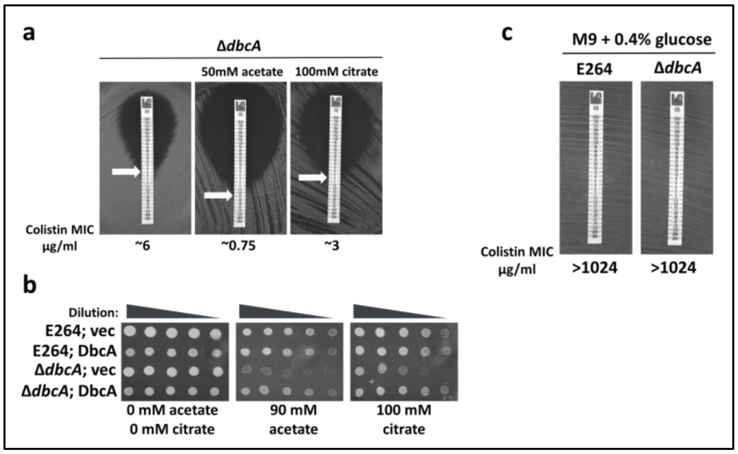

Supplementary Figure S4. Effect of acetate and citrate on colistin MIC of Δ*dbcA*, sensitivity of Δ*dbcA* to acetate or citrate and colistin MIC of *B. thailandensis* E264 and Δ*dbcA* on M9 growth media. **(a)** Further reduction of colistin MIC of Δ*dbcA* by acetate or citrate. Colistin MIC of Δ*dbcA* measured on MH2 agar media with supplementation of either acetate or citrate as indicated. **(b)** Sensitivity of Δ*dbcA* to acetate or citrate. 1:10 dilutions of indicated strains were spotted on MH2 agar plates with 100 µg/ml Tmp, 0.002% rhamnose and supplemented with acetate or citrate as indicated. Plates were analyzed after 48 hours of growth at 37°C. **(c)** Colistin MIC of *B. thailandensis* E264 and Δ*dbcA* on M9 growth media. The MIC was determined on M9 agar media supplemented with 0.4% glucose. Plates were analyzed after 48 hours of growth at 37°C.

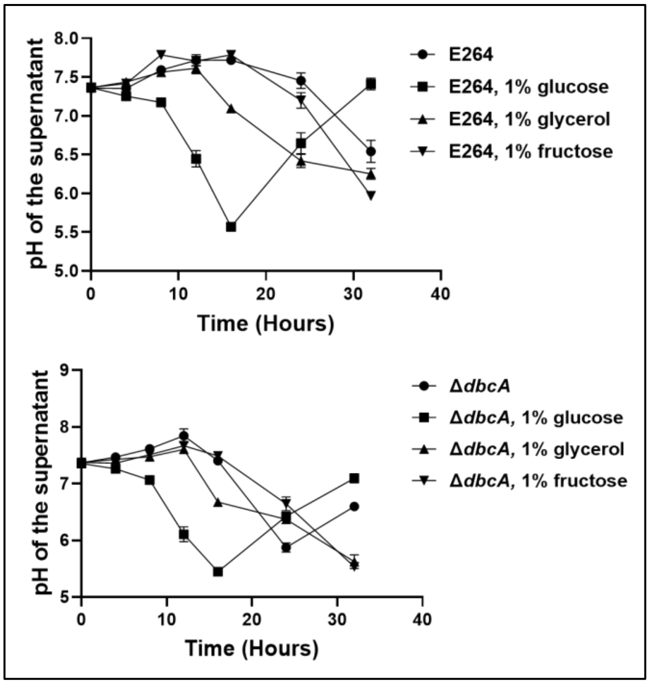

Supplementary Figure S5. Media acidification by *B. thailandensis* strains induced by glucose supplementation. 5 x 10^7 cells of *B. thailandensis* E264 and Δ*dbcA* were inoculated in 100 ml of MH2 media with 100 µg/ml Tmp and supplemented with either 1% glucose, 1% glycerol, or 1% fructose and grown for 32 hours at 37°C shaking at 225rpm. 1 ml was removed at each time point, centrifuged, and the pH of the supernatant was measured. The error bars indicate standard deviations of three biological replicates.

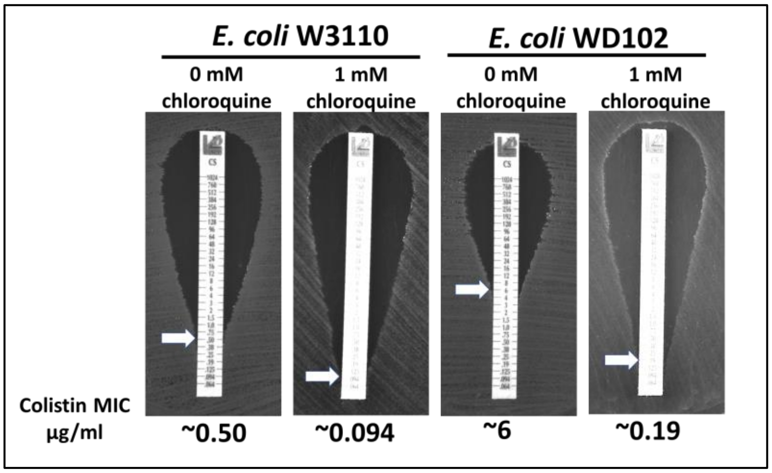

Supplementary Figure S6. Reduction of colistin MIC of *E. coli* strains by chloroquine. MIC was measured on LB agar media with and without chloroquine diphosphate salt as indicated. The initial pH was adjusted to pH 7.0 with NaOH since 1 mM chloroquine slightly decreased the pH of the media. Approximate MICs are denoted by white arrows.

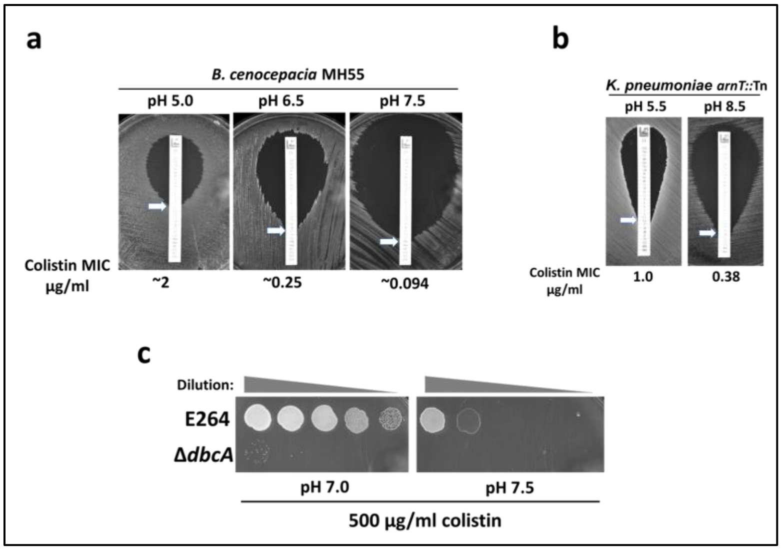

Supplementary Figure S7. Lipid A modification independent colistin resistance. **(a)** The colistin MIC was determined for *B. cenocepacia* MH55 on LB agar plates at different pH. **(b)** The colistin MIC for *K. pneumoniae arnT*::Tn [1] at pH 5.5 and pH 8.5 LB media. Approximate MICs are denoted by white arrows. **(c)** Spot assay of 1:10 dilution of *B. thailandensis* E264 and Δ*dbcA* at pH 7.0 and pH 7.5 LB media with 500 µg/ml colistin. The pH was adjusted with 100 mM Tris for pH 7.0, 7.5, and 8.5 whereas 100 mM MES was used for pH 5.0 and 6.5.

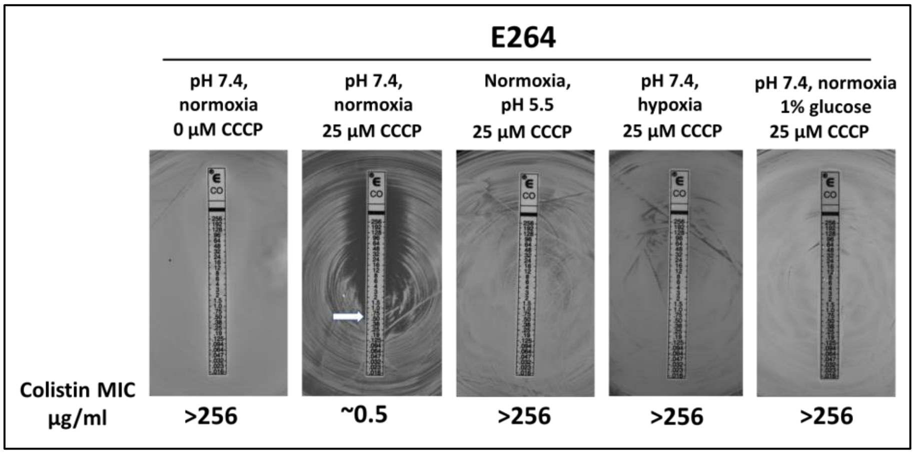

Supplementary Figure S8. The effect of acidic pH, hypoxia, or glucose supplementation in the reversal of the reduction of colistin MIC of *B. thailandensis* E264 by CCCP. MIC was determined using colistin E-test strips (Biomerieux). Arrow indicates approximate MIC.

Reference:

1 Ramage, B. *et al.* Comprehensive Arrayed Transposon Mutant Library of Klebsiella pneumoniae Outbreak Strain KPNIH1. *J Bacteriol* **199**, doi:10.1128/JB.00352-17 (2017).
